# A network of mixed actin polarity in the leading edge of spreading cells

**DOI:** 10.1038/s42003-022-04288-7

**Published:** 2022-12-07

**Authors:** Wen-Lu Chung, Matthias Eibauer, Wenhong Li, Rajaa Boujemaa-Paterski, Benjamin Geiger, Ohad Medalia

**Affiliations:** 1 Department of Biochemistry, University of Zurich, Winterthurerstrasse 190, 8057 Zurich, Switzerland; 2 Department of Immunology, and Regenerative Biology, Weizmann Institute of Science, Rehovot, 76100 Israel

**Keywords:** Actin, Electron microscopy

## Abstract

Physical interactions of cells with the underlying extracellular matrix (ECM) play key roles in multiple cellular processes. The actin cytoskeleton is a central driver and regulator of cellular dynamics, that produces membrane-protrusions such as lamellipodia and filopodia. Here, we examined actin organization in expanding lamellipodia during early stages of cell spreading. To gain insight into the 3D actin organization, we plated fibroblasts on galectin-8 coated EM grids, an ECM protein presents in disease states. We then combined cryo-electron tomography with advanced image processing tools for reconstructing the structure of F-actin in the lamellipodia. This approach enabled us to resolve the polarity and orientation of filaments, and the structure of the Arp2/3 complexes associated with F-actin branches. We show that F-actin in lamellipodial protrusions forms a dense network with three distinct sub-domains. One consists primarily of radial filaments, with their barbed ends pointing towards the membrane, the other is enriched with parallel filaments that run between the radial fibers, in addition to an intermediate sub-domain. Surprisingly, a minor, yet significant (~10%) population of actin filaments, are oriented with their barbed-ends towards the cell center. Our results provide structural insights into F-actin assembly and dynamic reorganization in the leading edge of spreading cells.

## Abstract (web summary)

Cryo-electron tomography reveals structural insights into F-actin assembly and dynamic reorganization in the leading edge of spreading cells.

## Introduction

Cell interactions with the extracellular matrix (ECM) play key roles in multiple cellular processes including tissue coherence and morphogenesis, cell migration, cell survival, and cytoskeletal organization^1–4^. Adhesive interactions with the matrix, primarily those mediated by integrin receptors, induce a local assembly of the actin cytoskeleton, that produces and coordinates both protrusive and contractile responses that drive cell motility^5^. Initial contacts between cells and the underlying ECM lead to progressive cell spreading, driven by major deformations of the membrane^6^. At the molecular level, the early integrin-mediated adhesive interactions initiate a complex cascade of cytoskeletal assembly and signaling events. Concertedly, they trigger radial cell spreading^7,8^, that is usually followed by cell polarization, manifested by the generation of a protrusive leading edge and a contractile trailing edge, at the interior and posterior aspects of the cell, respectively^9–11^.

Actin filaments polymerize at the leading edge and apply mechanical forces that drive the protrusive extension of the plasma membrane^12,13^. Together with diverse actin-associated proteins, they form a robust skeletal network that displays a retrograde flow, regulated by a fine balance between actin polymerization at the front and interaction with nascent matrix adhesions, at the lamellipodium-lamella junction^14–16^. Importantly, the Arp2/3 complex plays a key role in the formation and mechanics of the lamellipodium, by nucleating actin polymerization and branching^17–19^. The lamellipodium of adherent cells is a sheet-like protrusion that is typically hundreds of nanometers in thickness^20^. In migratory fibroblasts, the lamellipodium undergoes cycles of polymerization-dependent protrusion and actomyosin-driven retraction, that are believed to be regulated by variations in the mechanical load generated by the F-actin network^21–23^.

The organization of actin filaments within the lamellipodium was extensively studied by light and electron microscopy. These studies confirmed the presence of a branched F-actin network and suggested that the vast majority of actin filaments at the leading edge direct their barbed ends toward the plasma membrane^24–27^. However, these studies were mostly based on the use of permeabilized or fixed cell models that provided limited information concerning the precise filament organization and polarity.

The mode of cell spreading on the ECM is profoundly affected by the molecular composition of the underlying matrix and the set of adhesion receptors the cells possess^28,29^. Commonly, cell adhesion and spreading studies were conducted using specific adhesive proteins such as fibronectin, vitronectin, collagen, and laminin^30–33^. Each of these proteins may present different chemical and physical properties, interact with a different set of membrane receptors, and thus display distinct effects on cell spreading and motility. Distinct adhesive features were reported for members of the galectin family^34^, comprising several adhesive galactoside-binding animal lectins, that interact with a variety of cell-surface glycans. They are expressed in a variety of tissues and were shown to affect cell-ECM and cell–cell adhesion, trans-membrane signaling, cell spreading, and cell migration^35^. Most importantly, galectins contribute to cancer progression by regulating the migration and cell adhesion properties of tumor cells^36^.

Within the galectin family, the dimeric galectin-8 (gal-8) was shown to play an important role in platelet activation and angiogenesis^37^, and induce fast and efficient spreading of cells^38^. Gal-8 is expressed and secreted from cells to the ECM in health and disease. For example, elevated levels of gal-8 correlated with cartilage degeneration, suggesting that gal-8 serves as a functional disease marker in human osteoarthritis^39^. Galectins interact with the cell-surface receptors that impact cell signaling and adhesion to the ECM. These interactions facilitate cell migration in a variety of model systems such as glioblastoma^40^, keratinocyte, B16 melanoma and act through the Rho/Rac pathway in different cellular systems^41,42^.

It was recently demonstrated that cell adhesion to gal-8-coated surface leads to essentially continuous spreading dynamics, unlike spreading on fibronectin which consists of protrusion-and-retraction cycles^43^. Consequently, the average projected area of fully spread HeLa cells on gal-8 is about twice larger than the projected area of the same cells plated on fibronectin^16^. Notably the initial spreading of the cells on gal-8 is radial, forming thin lamellipodia with a typical thickness of 100–150 nm, that is optimal for cryo-electron tomography (cryo-ET) analysis without altering the cellular integrity, or applying chemical fixation^43^. This feature and the uninterrupted nature of the protrusive expansion on gal-8 enabled us to retrieve fundamental information on the assembly of the actin network in the expanding lamellipodium.

Here, we applied cryo-ET to mouse embryonic fibroblasts (MEFs) plated on gal-8-coated EM grids, enabling us to analyze the structure of the actin network at the cell expanding edges in cellulo. We further used a combination of image processing approaches to determine quantitatively the polarity of individual actin filaments located at the protruding lamellipodium. These analyses revealed unexpected variations in the actin directionality that define functional sub-domains within the protruding lamellipodia of spreading cells, including a significant level of filaments that are oriented their barbed ends towards the cell center. These findings suggest that rapid reorganization of the actin network architecture occurs within the protruding leading edge of cells spreading on galectins.

## Results

The 3D organization of the cells, including the interface between the actin cytoskeleton and the plasma membrane, was previously visualized by cryo-ET^44^. However, a major limitation in this approach is the thickness of spreading cells, which hinders the spatial resolution and the precise localization of the cellular processes. This limitation can be overcome by allowing cells to spread over gal-8-coated surfaces which markedly reduces the thickness of the adhering cells^38,43^. It was demonstrated that the presence of gal-8 in the ECM correlates with cancer and metastasis^45^, and increases cell growth and adhesion of metastatic cells^46^.

### In cellulo structural analysis of actin and Arp2/3

**Fig. 1:**
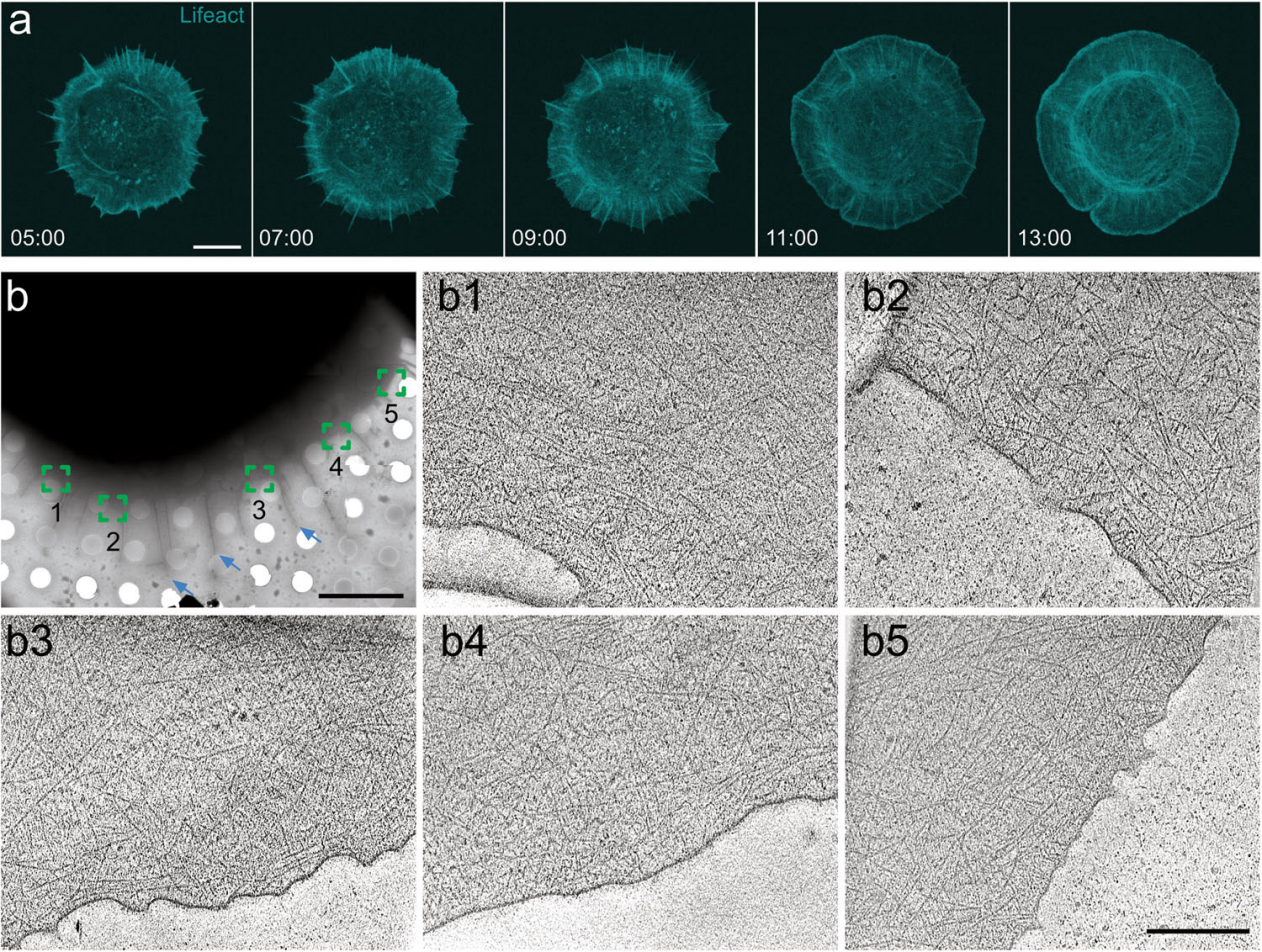
Cell spreading on galectin-8 coated substrate. **a** Time-lapse images of a MEF cell transfected with Lifeact-mRuby let to spread on gal-8-coated substrate (Supplementary Movie 2). Scale bar: 10 μm. **b** A cryo-EM image of a representative MEF cell spread on a gal-8-coated EM grid. Scale bar: 8 μm. The area subjected to cryo-ET analyses indicated in boxes. A representative x-y slice through each tomogram in shown (**b1**–**b5**). Filopodia are indicated with cyan arrows. Scale bar: 300 nm.

MEFs cultured on gal-8-coated surfaces exhibited radial membrane protrusions within 20 min after contacting the coated surface (Fig. 1a and Supplementary Movie 1 and Movie 2). The spreading dynamics were significantly faster and continuous, compared to those obtained with other ECM proteins, e.g., fibronectin^43^. However, the characteristic actin dynamics and treadmilling are still detected (Supplementary Movie 2). Moreover, both Arp2/3 and F-actin showed a conspicuous association with the wide annular leading edge (Supplementary Fig. 1). In this study, we conducted experiments with cells growing on gal-8-coated EM grids that were vitrified within 10–20 min after the cells were engaged with the EM grids.

Low magnification cryo-EM imaging revealed radial spreading cells with both lamellipodial and filopodial protrusions (Fig. 1b). We primarily focused on the edges of the circumferential lamellipodia and acquired multiple tomograms. Figure 1-b1 to 1-b5 shows typical positions that were analyzed by cryo-ET. The thickness of the 57 acquired tomograms was 102 ± 25 nm, which provided us with an opportunity to systematically acquire high-quality data of essentially an entire peripheral ~600 nm wide belt of the lamellipodium edge. Notably, when spreading on fibronectin-coated grids, cells presented edges that are significantly thicker, typically ~200 nm^32,47^.

**Fig. 2:**
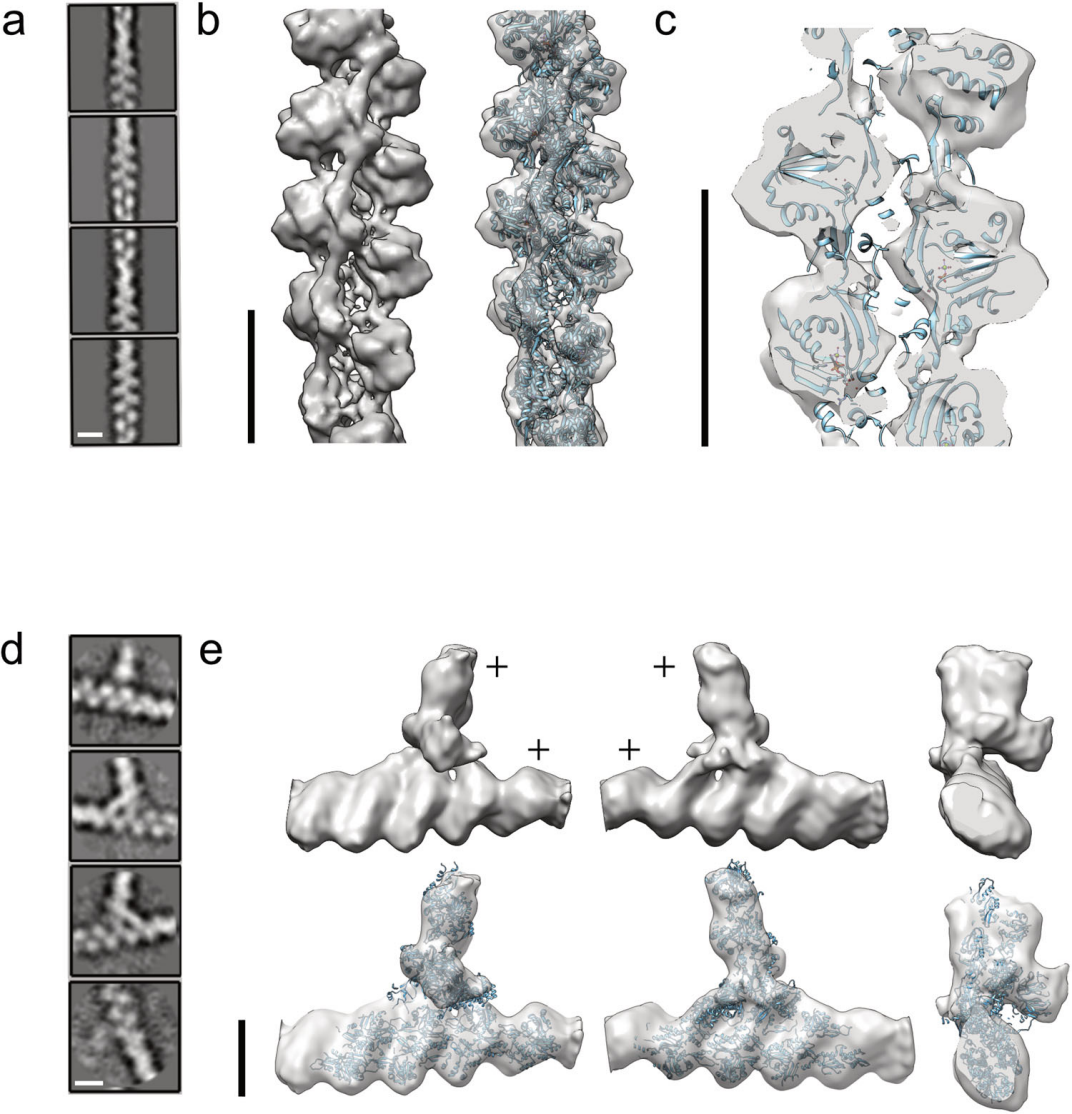
Three-dimensional reconstruction of actin and the Arp2/3 complex. **a** Representative 2D class averages of actin segments, 36 nm in length, analyses from cryo-tomograms of spreading cells. **b** The structure of actin filaments resolved to 14.4 Å (Supplementary Fig. 4a). Cryo-EM structure of actin EMD-15106 was docked into the structure reconstructed from in situ cryo-tomograms. **c** A cutaway view through the in situ actin structure indicates the quality of fitting of the in vitro structure. **d** 2D class averages of Arp2/3-mediated branches, identified by template matching (see methods section). **e** Reconstructed structure of actin branches at 26 Å resolution (Supplementary Fig. 4b). The direction of actin barbed end is labeled with ‘+’. The structure of the extracted branches (EMD-11869) was docked into the in situ reconstructed structure (lower panel). Scale bar: 8 nm.

Initially, actin filaments in 7 tomograms were manually segmented (Supplementary Fig. 2) and used for training a convolutional neural network^48^, enabling us to automatically segment actin filaments from additional 50 tomograms. By applying the actin polarity toolbox (APT)^49^, we extracted subtomograms, 36 × 36 × 36 nm^3^ box size, and reconstructed the structure of the actin filament at 14.4 Å resolution (Fig. 2a–c and Supplementary Fig. 4a) determined by Fourier shell correlation to EMD-15106^50^. The 3D refined actin filament structure was used to determine the filament polarity throughout the analyzed volume.

The branched actin network of the lamellipodia is decorated by the Arp2/3 complex, which is found at branched junctions along the actin filaments^51^. The Arp2/3 comprises the two actin-related proteins 2 and 3, Arp 2 and 3, in addition to ArpC 1, 2, 3, 4, and 5^52^. This heptameric complex was previously resolved at 9 Å resolution using subtomogram averaging from cytoskeleton extracted cells^53^, and at a more modest 32 Å resolution in situ^54^. Here, we utilized a template matching approach for localizing the complex in 96 tomograms (Supplementary Fig. 3a). We extracted subtomograms using a 31.8 × 31.8 × 31.8 nm^3^ box size, and applied 3D-subtomogram averaging. Figure 2d shows class averages of projection images at different orientations with respect to the x-y plane, suggesting a reasonable orientation coverage. We obtained a refined 3D structure with a gold-standard resolution of 26 Å (Supplementary Fig. 4b) by using 6149 subtomograms. We were able to dock the Arp2/3 structure EMD-11869^53^ into our structure (Fig. 2e) with a cross-correlation coefficient of 0.84 analyzed with UCSF Chimera^55^. The in situ structural analysis of both actin filaments and the Arp2/3 allows mapping these structures back into the reconstructed cellular volumes and investigating the organization of the lamellipodial actin network in high detail.

### Mapping of actin filament’s orientation and polarity in the lamellipodia of spreading cells

**Fig. 3:**
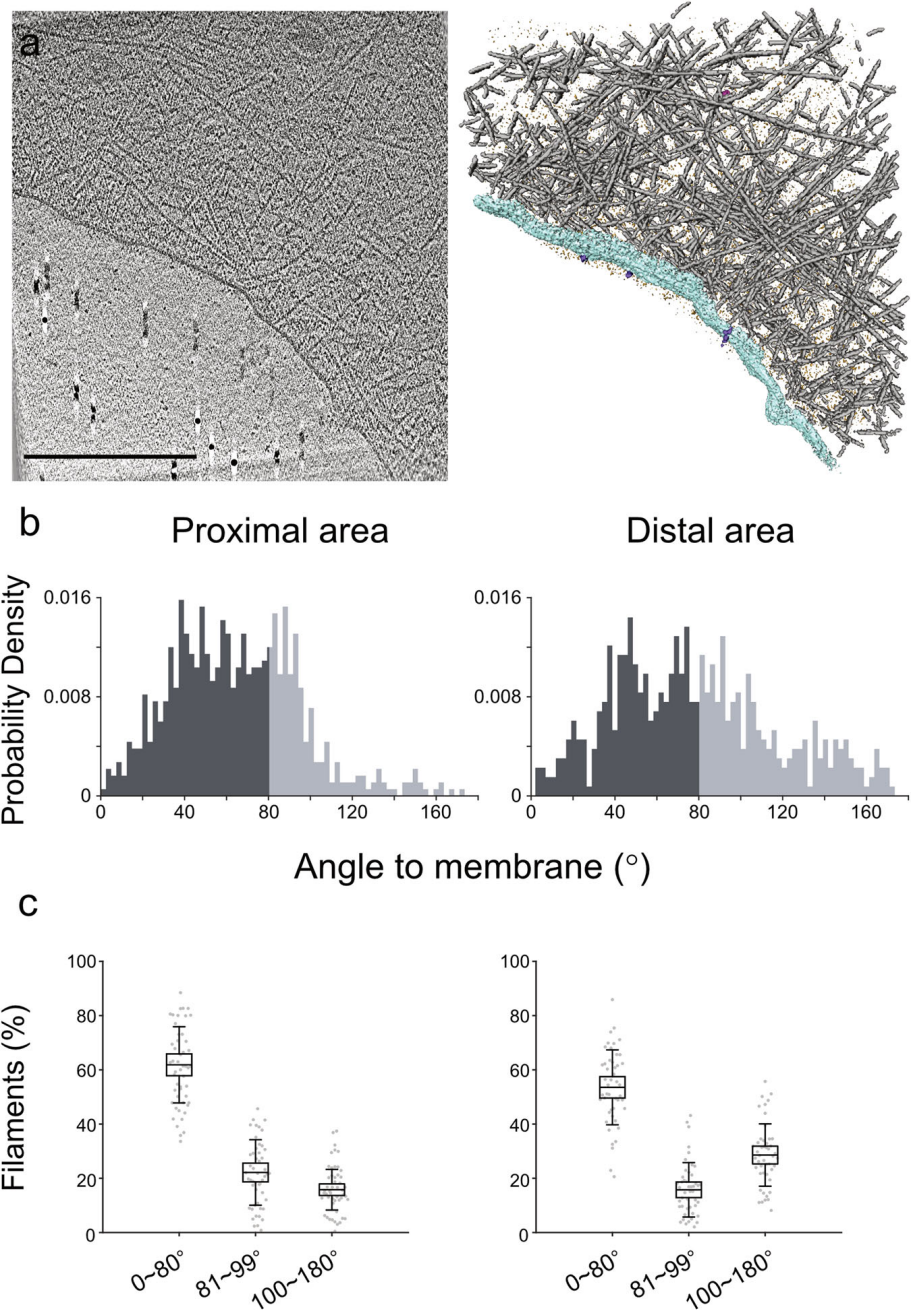
Filament network and filament-membrane orientation. **a** An x-y slice through a cryo- tomogram (left) and its rendered isosurface view of a lamellipodium protrusion. Actin filaments (gray), cell membrane (turquoise), membrane receptors (purple), and cell components (magenta). Scale bar: 300 nm. **b** The orientation of filaments with respect to the membrane was measured in 47 tomograms at the membrane proximal area of the cells (0–40 nm away from membrane) and distal area (360–400 nm away from membrane). The dark gray region represents filaments that contain vectorial components with the barbed end towards the membrane. **c** The boxplots showing the proportion of the different filament directionalities with respect to the membrane, with 1 SD and 1.96 SEM in whiskers (*N* = 47 tomograms).

The 3D network geometry of actin cytoskeleton affects the overall mechanical properties of cellular processes, using the asymmetrical polymerization dynamics of actin. The thermodynamic polarity of actin filaments, which relies on the existence of a fast-growing barbed end, plays a key role in regulating the dynamics and mechanics of the whole network. Developments in cryo-EM and tomography, in conjunction with novel image processing approaches, allow a high-enough resolution to determine the precise polarity of individual actin filaments within the lamellipodial network. Initially, we visualized the complete organization of the actin filaments by isosurface rendering of the tomograms (Fig. 3a, Supplementary Movie 3, and Supplementary Fig. 2). This initial step provided the basis for comprehensive structural analysis of the orientation and polarity of the filaments within the tested volumes relative to the membrane. Towards this end, we mapped back the structure of the actin filaments to obtain the polarity information of each filament as reported (refs. ^49,56^, Methods section). We then quantified the relative angle between each actin filament and the closest plasma membrane by comparing the polarity of the filament to the normal angle of the nearest plasma membrane.

Initially, we compiled all the data and analyzed all filament-membrane orientations along the protruding membrane and with 40 nm slabs away from the membrane towards the cell center. The barbed end defines the representative coordinate of each filament (a polarity perpendicular and towards the plasma membrane is defined as 0°). In the membrane proximal area (0–40 nm away from the membrane), more than 60% of the filaments have their barbed ends towards the membrane (Fig. 3b, left, dark gray). While in the membrane distal area (360–400 nm away from the membrane and towards the cell center), we found that 55% of the barbed ends are pointing towards the membrane (Fig. 3b, right, dark gray). Respectively, the density of the actin changes only moderately within the analyzed 400 nm distance from the membrane, with the highest filament density at ~80 nm away from the membrane, (Supplementary Fig. 5a). The barbed end density shows a slight increase close to the membrane, presumably due to the higher nucleation activity at the membrane proximal area (Supplementary Fig. 5b). This shows that when merging data from a large number of lamellipodial volumes, neither the density nor the directionality of the actin seemed to change across the network from the membrane towards the cell body. Thus, in such an analysis, radial and circumferential variations of actin density and polarity of potentially different network states may be averaged and hindered.

The actin cytoskeleton at the lamellipodia is branched by the Arp2/3 complex. In the acquired data, we found an Arp2/3 density of 2500 µm^−3^. Overall, the branched actin network showed a 1:3 ratio between the number of Arp2/3 and actin filaments (Supplementary Fig. 6a), suggesting that a significant number of actin filaments in the studied volumes were not branched.

### Radial diversity of filament orientations in the lamellipodia of spreading cells

**Fig. 4:**
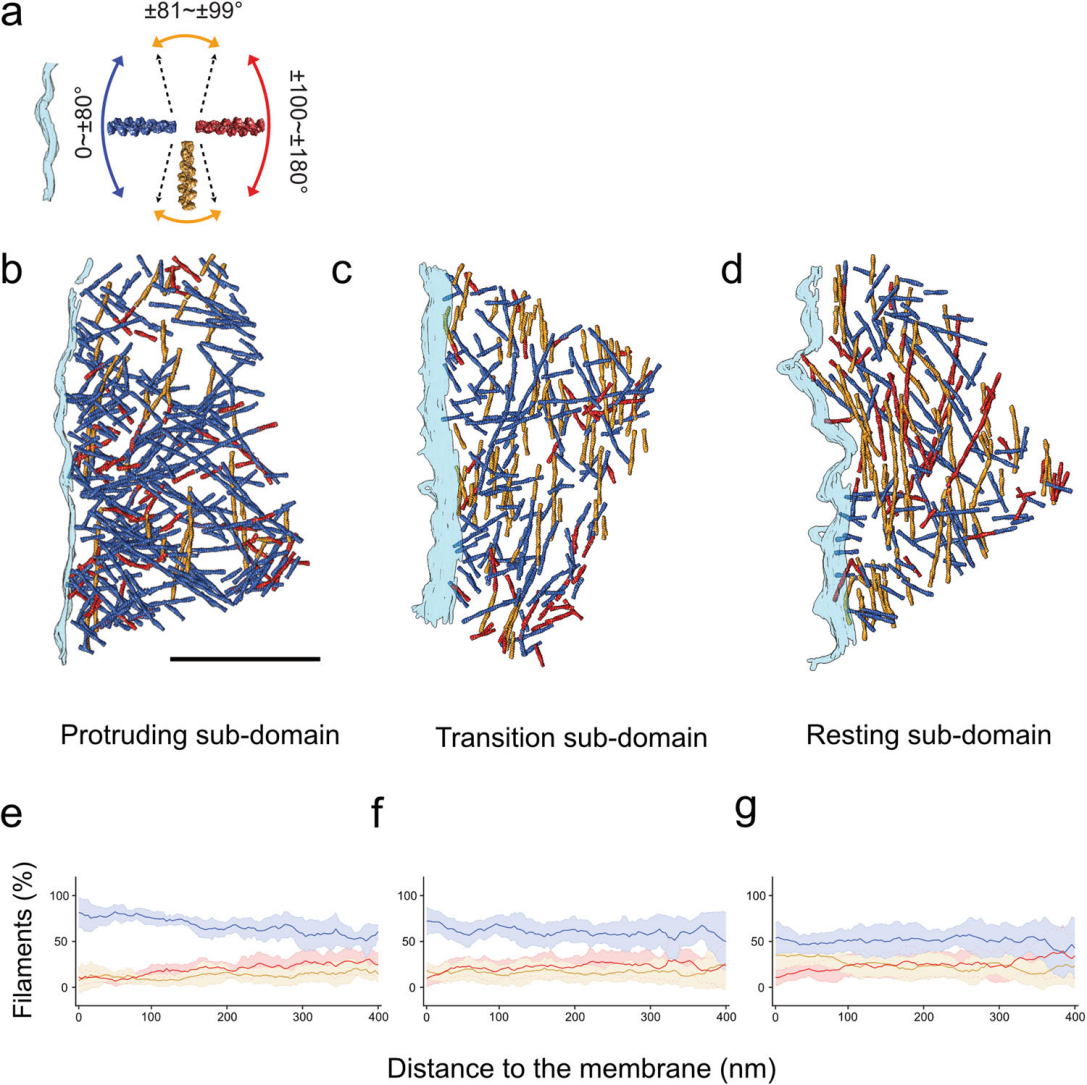
Three different sub-domains within lamellipodia. **a** A color scheme indicating the angles between actin filaments and plasma membrane (turquoise). **b**–**d** Surface rendered views of representative tomograms from the 3 different sub-domains with the actin filaments colored according to their orientation with respect to the plasma membrane. Scale bar: 300 nm. **e**–**g** The fraction of the forward (blue), parallel (mustard), and backward (red) actin orientations as a function of the distance from the membrane are plotted as continuous lines, with a shaded error bar of data standard deviation, for protruding, transition, and resting sub-domains (*N* = 10, 16, 21 tomograms).

To understand the organization of actin filaments and their orientation relative to the plasma membrane, we focused on the 400 nm layer of the lamellipodial actin located underneath the plasma membrane of spreading cells, and analyzed the polarity of individual filaments as well as the 3D organization of the network. We found that the barbed ends of 60% of the filaments are oriented towards the membrane (forward orientation), 20% exhibit a parallel-filament orientation (defined as being largely parallel to the plasma membrane edge), while ~10% point towards the cell interior (backward orientation) (Fig. 3c). We have defined the angular range of the three-characteristic orientations of actin filaments, relative to the protruding plasma membrane, as follows (Figs. 3c, 4a): ‘forward filaments’ are oriented at an angle of 0–80° (with their barbed end towards the membrane) and shown in blue, the parallel filaments are oriented at an angle range of 81–99° (mustard), while the ‘backward filaments’ are oriented at an angular range of 100–180° (red). This color code allows for an easier appreciation of the overall network architecture while preserving the accurate orientation of individual filaments. We then compared the proportion of each of the three filament categories populating the first 400 nm layer underneath the cell edge to identify the state of the lamellipodia.

Further confirmation of the assignment of actin polarity provided by the comparison of the filament-to-membrane angle distribution of all the actin filaments to the distribution of Arp2/3, while each Arp2/3 is back-mapped from the averaged structure and assigned as a mother filament and a daughter filament. Both of Arp2/3 orientation and actin showed a similar angle distribution at around 80° (Supplementary Fig. 6c). By visualizing the branched network of Arp2/3 and actin filaments (Supplementary Fig. 3b), we confirmed that most actin filaments found in the Arp2/3 matches the known orientation of actin filaments.

### Circumferential diversity of filament orientations in the lamellipodia of spreading cells

Next, we tested the circumferential variability of actin filament orientations along the expanding cell front. Our analysis revealed three main sub-domains within the ~400 nm wide belt of the spreading lamellipodium. Each sub-domain displayed a distinct pattern of actin organization. Based on the relative prominence of the forward actin filaments in these sub-domains we refer to them as the ‘protruding’, ‘transition’, and ‘resting’ sub-domains (Fig. 4b–d, Supplementary Fig. 7). The assignment of the sub-domain are neither biased by proximity to filopodia protrusions nor can be predicted by the precise orientation of the plasma membrane (Supplementary Fig. 8). In the protruding sub-domain, the membrane proximal area exhibits the highest portion (around 85%) of forward-oriented filaments (Fig. 4e). The level reduced to about 60% in the most distal area (located at 360–400 nm away from the membrane) (Fig. 4e). Interestingly, the parallel filaments display a comparable level in the proximal and distal areas of the protruding sub-domain, while the levels of backwards-filaments gradually increased from ~10% in the proximal area to ~30% in the distal area from the membrane (Fig. 4e).

In the ‘transition sub-domain’ (Fig. 4c), the network shows variable levels of forward filaments (in the range of 70% to 50%) that hardly correspond to their distance from the plasma membrane (Fig. 4f). Here, the prominence of the parallel filaments is higher than that detected in the protruding sub-domain, suggesting that while the network may still be protrusive, the parallel network and the backward filaments may support the establishment of contractile actomyosin force generation. This is more pronounced in the resting sub-domain, where around 40% of the actin filaments are parallel to the membrane (Fig. 4d, g). Interestingly, the polarity of actin filaments along the thickness of the lamellipodia and as a function of the distance from the plasma membrane, resemble the organization discussed above (Fig. 4), for the three sub-domains (Supplementary Fig. 6d).

**Fig. 5:**
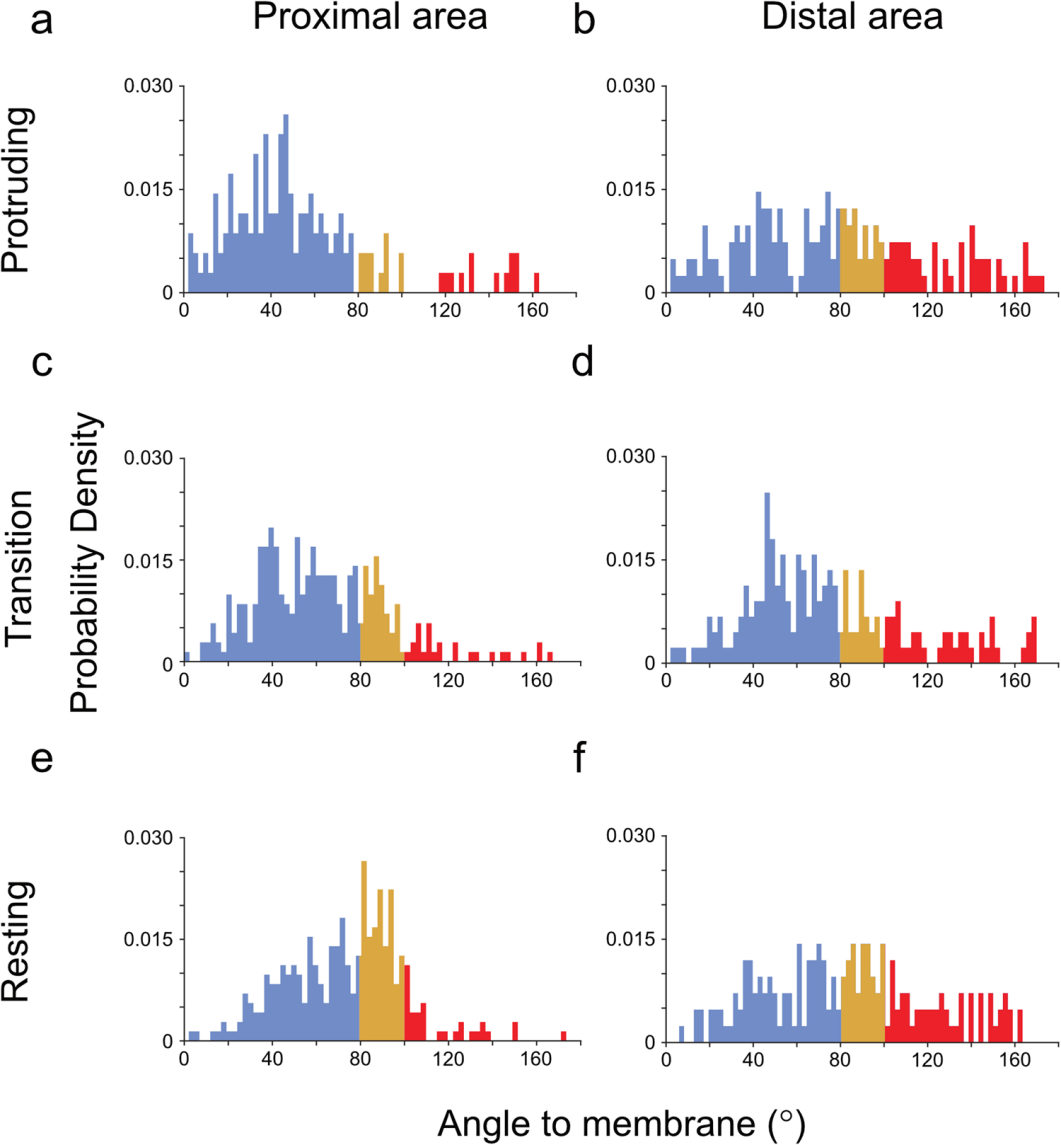
The distribution of filament orientations in the three different lamellipodia sub-domains. The orientation of actin filaments in respect to the plasma membrane is shown. The histograms show actin filament orientation at the membrane proximal regions and distal regions within the lamellipodia. Protruding (**a**, **b**), transition (**c**, **d**), and resting sub-domains (**e**, **f**). The filament-membrane orientation distribution at the membrane proximal (left) and distal areas (right). The colors indicate the angular orientation as shown in Fig. 4a.

A detailed comparison of actin directionality at the proximal and distal area (40 nm and 400 nm away from the membrane, respectively) is shown in Fig. 5. Remarkably, in the protruding areas, the angle distribution of filaments at the proximal and distal areas vary significantly and broaden at the distal areas (Fig. 5a, b). In the proximal area, the highest prominent angle is ~40°, while an almost even distribution ranging between 20 and 140° is found at a distance of 400 nm from the membrane. In the ‘transition sub-domains’, a broader distribution of actin directionality is found at the proximal and distal area of the lamellipodia (Fig. 5c, d). A subpopulation of the filaments is aiming at the membrane with an angle of ~40° while a similar proportion of actin is oriented between 80–90° (parallel orientation). Such an orientation has been suggested to support less protruding leading edge and allow a dynamic growth of actin filaments that would support the emergence of a protruding network^23,57^. In contrast to the ‘protruding’ and ‘transition’ sub-domains, the proximal area of the ‘resting’ sub-domain (Fig. 5e) is characterized by the highest frequency of parallel filaments. Surprisingly, throughout all sub-domains, we have found that a similar portion of backward-oriented actin filaments with a slight increase in the distal area.

## Discussion

The reduction in lamellipodia thickness, induced by substrate-immobilized gal-8, allowed us to apply cryo-electron tomography for studying the molecular architecture of the actin cytoskeleton in the leading edge of intact spreading cells, at relatively high resolution. Gal-8 has been shown to be a constituent of the ECM, functions as a pro-metastatic agent. It was suggested that it functions via enhanced cell adhesion properties^42^. The spreading of cells on a typical ECM protein, e.g., fibronectin, proceeds as a mix of protrusive activity and retraction^43,58,59^. Thus, throughout the cell spreading process, different regions at the cell periphery may undergo unsynchronized protrusion-retraction transitions, rendering it challenging to generate a reliable correlative analysis of actin organization in protruding lamellipodia. In previous studies, it was shown that during early stages of cell spreading on gal-8 coated support, the protrusive activity of nearly the entire lamellipodium is continuous and is not interrupted by retractive phases^43^, which enables us to study protruding lamellipodium by cryo-ET. Moreover, the thickness of the lamellipodium in cell spread on gal-8 is 80–150 nm, which enables us to resolve actin filament orientation without fixing or extracting the cells, as commonly practiced in previous studies^17,26,27^.

**Fig. 6:**
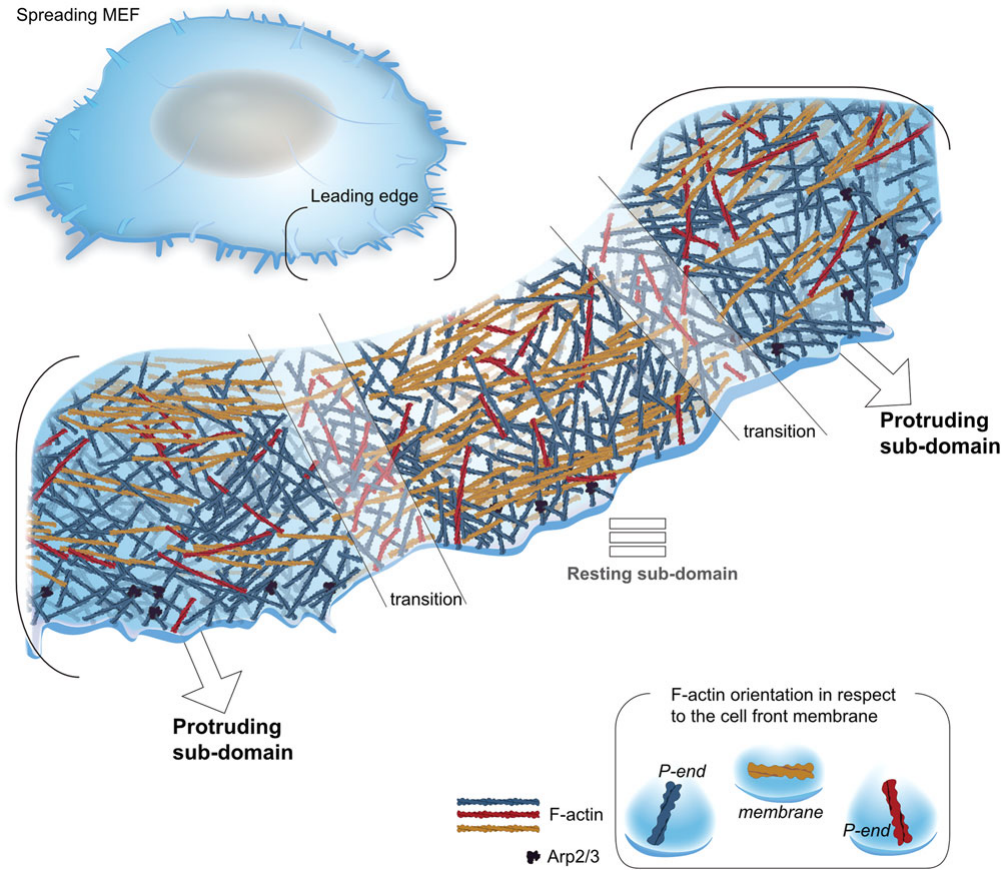
The lamellipodia architecture in spreading cells. A model depicting the actin organization at the edge of a spreading cell. Three distinct actin sub-domain exhibit different directionality of actin filaments were identified. A protruding sub-domain in which the majority of the actin filaments have their barbed ends pointing towards the membrane (blue), while parallel filaments (mustard) and filaments that are pointing towards the cell body (red) can be detected as well. In the resting sub-domain, less filaments are directed towards the membrane while more parallel filaments are detected. Transition sub-domains are spaced between the protruding and resting sub-domains. In all the three network architectures, parallel oriented actin filaments (mustard) are enriched in the resting sub-domains although they can also be seen in transition and protruding sub-domains. Similar amounts of filaments that are pointing towards the cell body (red) were detected in all sub-domains.

Applying subtomogram averaging, we reconstructed the structure of the Arp2/3 complex, in situ, and analyzed in detail the precise orientation and polarity of the actin network throughout the leading edge. This analysis revealed a heterogenous cytoskeletal organization, manifested by the presence of distinct structural sub-domains with different F-actin orientations, as summarized in Fig. 6. We propose that different actin organizations, both along the “radial axis” (proximal-distal, relative to the cell edge) and along the leading edge (“circumferential axis”) reflect aspects of the actin network dynamics. For example, there are regions in which the actin barbed ends are primarily directed towards the membrane (we refer to these regions as protruding sub-domains) while in neighboring regions there are prominence of filaments that run parallel to the plasma membrane, which we refer to as “resting sub-domains”. Additionally, the transition sub-domains, containing smaller fraction of parallel filaments than in the resting sub-domain, likely corresponding to a transition state between protrusive and resting states. Surprisingly, in essentially all analyzed sub-domains, ~10% of the filaments are oriented with their barbed end towards the cell center, rather than the expected opposite orientation towards the plasma membrane^24,27,60–62^. Filaments with their barbed end towards the cell center may be involved in the establishment of nascent adhesions that can further develop into focal adhesions, allowing the cell to use actomyosin system to transport cargo away from the leading edge. Although the precise function of the latter actin filaments is to be uncovered, they may be originated out of stochastic polymerization events that occur at the cell edge.

The variations in filament organization were most pronounced along the radial axis, comparing regions that are located close to the protruding edge, to those located 300–400 nm away into the cell center. As indicated, the differences were primarily manifested by the relative prominence of forward filaments oriented with their barbed ends towards the membrane along the radial axis^23,57,60–62^. Notably the three sub-domains are in general agreement with previous reports which analyzed the actin network in leading edge of fish keratocytes and MEF cells^23,27,57^. The data showed how changes in angles between actin filament and the plasma membrane can alter the mechanical load onto the network. Specifically, larger angles were shown to decrease the load and eventually lead to reduce cell movement. Thus, filaments that encounter the membrane at larger angles might lag behind neighboring filaments that elongated centripetally toward the membrane. These stalled filaments may eventually detach from the membrane and produce a membrane parallel-orientated filaments. Indeed, differentially oriented populations of actin meshwork were proposed to affect protruding and resting lamellipodia^11^.

Our current observation demonstrates a molecular heterogeneity in actin filament orientation and polarity, suggesting that within the lamellipodia of spreading cells there are submicron scale domains with distinct dynamic properties. This is inconsistent with the view that actin polymerization in the leading edge is driving a seemingly uniform and coherent retrograde flow. It is unclear how individual filaments are changing their orientation while moving centripetally. Yet, it appears likely that molecular interactions of individual actin filaments with nearby immobile components, such as nascent focal complexes and diverse cytoskeletal complexes, introduce local perturbations to the retrograde flow, which create and reinforce the local heterogeneity at and near the leading edge. Such interactions of actin filaments may be fundamental for force generation that drive both the protrusion of the leading edge and the maturation of nascent integrin adhesions into mature focal adhesions^63–65^. The typical average flow rate, as measured for example by direct fluorescence speckles microscopy^66^, is in the order of 0.5–0.8 µm/min. However, the rate of this flow may vary spatially and temporally, due to local rates of polymerization, and the constant change in filament orientation. This may be too small and transient to be detected using live cell imaging. Recent in vitro and in situ experiments demonstrated how actin meshwork flowing over VBS1-activated vinculin can attenuate the flow, resulting in massive active bundling^67,68^. It is likely that actin filaments, which encounter such local perturbations in live cells, undergo radical changes in their orientation while translocating from the ‘proximal zone’ (0–40 nm on the leading edge) to the ‘distal zone’, located ~300–400 nm towards the cell center. As indicated within this timeframe, along the radial axis, there is a decline in ‘forward filaments’ and an increase in the prominence of backward and parallel filaments.

A key component of the lamellipodium actin network, the Apr2/3 complex forms the branched actin filament arrays^69^. Our findings, which are supported by previous observations, suggest rather low frequency of Arp2/3 branching as J.V. Small and colleagues measured the Arp2/3 branches in lamellipodia of fibroblasts^26^. Using electron tomography of detergent-treated and stained cells, they evaluated the branch density to one every 0.8 μm filament length. In this study, based on an unambiguous localization of Arp2/3 in intact cells, we detected genuine Arp2/3-mediated branches on only a fraction of the actin filaments within the lamellipodium, which translates to a branch every ~0.6 μm of filament length. The limited signal-to-noise ratio in cryo-ET may result in artificial gaps along segmented actin filaments. Therefore, the precise continuity of all filaments is challenging to follow. However, the rather large statistics used in this study and the detected high frequency of Arp2/3 in comparison to branches in lamellipodia of fibroblasts, suggest that the measured filaments to Arp2/3 ratio is reliable.

Since Arp2/3-mediated branch density affects the actin stiffness and is suggested to play a central role in lamellipodia protrusion, the density of branches may vary according to the cell type during faster or slower protruding processes^70,71^. Based on these findings, we propose that structural fluctuations in protruding regions within the lamellipodia edge, may represent macroscopic manifestations of local variability in filament orientation and branching. This proposal is in agreement with previous studies suggesting rather low frequency (yet significant and reproducible) of branches in protruding leading edges of fibroblasts^26^.

The results presented here evaluate  the significant cytoskeletal characteristics during cell spreading. With implications on cell migration, it is noteworthy that the spreading of cells on gal-8-coated substrates is continuous and overall faster than that on fibronectin which consists of repeated cycles of protrusions and retractions^43^. Cell spreading and motility require the reorganization of the branched actin network into mixed polarity parallel bundles, a process that involves focal adhesion localized actin binders, such as vinculin and α-actinin^67,72^. The diverse actin orientations, which include actin directed with their barbed ends towards the cell center, create mixed polarity actin organization that allows an efficient establishment of contractile actomyosin complexes. Similarly, mixed polarity of actin was found in protruding platelets pseudopods^56^.

## Materials and methods

### Cell culture and sample preparation

MEFs expressing vinculin-venus^49,73,74^ were used for optimal adhesion and spreading on EM grid. The cells were cultured in Dulbecco’s Modified Eagle’s Medium (Sigma–Aldrich, D5671) with 10% fetal bovine serum (Sigma–Aldrich, G7524), 2 mM L-glutamine (Sigma–Aldrich, G7513), and 100 mg/ml penicillin-streptomycin (Sigma–Aldrich, P0781) at 37 °C and 5% CO_2_. Glow-discharged EM grids with carbon support film (R2/2,Au mesh;Quantifoil, Jena, Germany) were coated with 25 mg/ml gal-8, 2 h room temperature. The cells were detached with 5 mM EDTA, resuspended in serum-free medium, and seeded onto the EM grids. The cells were then incubated at 37 °C and 5% CO_2_ for 10–20 min to allow cell spreading. Before plunge-freezingin liquid ethane, the grids were washed with 1× PBS (Fisher Scientific, BP399-1), and 4 ml of BSA-coated 10 nm fiducial gold markers (Aurion, Wageningen, Netherlands) was applied onto the grids.

### Immunofluorescence (IF) and live cell imaging

For live cell imaging, cells were plated at a density of 5 × 10^4^ cells ml^−1^ onto the 35 mm cell culture dish with 14 mm diameter glass bottom (MatTek, catalogue number: P35G-1.5-14-C) coated with Gal-8. Video recordings started 2–5 min after the cells were added to the dish. Interference reflection microscopy (IRM) time-lapse imaging were carried out using the DeltaVision RT microscopy system (Applied Precision Inc., Issaquah, WA, USA), equipped with a ×100 oil immersion objective (1.3 NA, UPlanSApo), at 15 s time intervals between frames. Confocal images and videos were taken with ANDOR Dragonfly spinning disk confocal microscope using ×100 objective and an sCMOS (Zyla) camera. The transfections were done with jetOPTIMUS^®^ (Polyplus-transfection^®^ SA), following the manufacturer protocols.

For IF, cells were plated on Gal-8 coated cover-slip and let spread for 16 min before fixation with 3.7% paraformaldehyde. After washed and permeabilized with 0.1% Triton-X/PBS for 3 times/ 5 min, cells were blocked with 1% BSA in 0.1% Tween-20/PBS for 1 h. Cells were then stained with 1:100 Anti-p34-Arc (Sigma–Aldrich, 07-227) for 1 h. After three times wash of 5 min with 0.1% Tween-20/PBS, cells were stained with 1:400 goat anti-rabbit IgG (Alexa Fluor® 488, Abcam) and 1:100 Alexa Fluor® 647 phalloidin for 1 h. Before imaging, coverslips were mounted with fluorescence mounting medium (Dako Omnis). Confocal images were taken with Olympus IXplore SpinSR10 super resolution imaging system equipped with two sCMOS cameras, and processed with ImageJ^75^.

### Cryo-electron tomography

Titan Krios G2 transmission electron microscope (Thermo Fisher Scientific, Waltham, MA) equipped with energy filter and K2-summit direct election detector (Gatan, Pleasanton, CA) was used for data acquisition. The microscope was operated at 300 keV in zero-loss mode; energy filter slit width was set to 20 eV. The microscope was controlled by SerialEM^76^. Tilt series were acquired ranging from −60 to 60° with 3° increments at −4 μm defocus. The tilt series were acquired at dose-fractionated mode with a frame rate of 0.2 sec/frame to a total of 1.2 sec/projection. The magnification was ×64,000 resulting in a pixel size of 2.207 Å with an accumulated electron dose of around 100 e^−^/Å^2^. The tomograms were reconstructed using the TOM toolbox^77^ and CTF corrected^78^. The data were collected from five different batches of experiments and at least 4 cells of each batch.

### Actin filaments polarity determination and 3D reconstruction

We followed the APT framework for filament polarity determination^49^. In brief, 212,537 actin segments were extracted from 58 tomograms with an equidistant spacing of 11 nm along the filaments. The filaments were segmented manually with AMIRA (Thermo Fisher Scientific, Waltham, USA) and then automatically using a convolutional neural network^48^. The automated segmentation was further processed in UCSF Chimera^55^ using the hide dust command and trimming the volumes to reduce false positive labeling. The subtomograms box size was set to 36 nm in length. Next, the central 11 nm of each subtomogram were projected prior to 2D classification (Fig. 2a) and 3D reconstruction by RELION^79^. For the final reconstruction of F-actin, 149,617 particles were selected and 3D refined, prior to the polarity determination.

To determine the polarity of the actin filament network, we adapted the APT for dendritic actin networks and the determination of filament-membrane orientation. In each tomogram, actin filaments were defined as ${F}_{j}^{i}$ with $i$ the filament number and $j$. the segment number within the filament. Actin filaments are generally capped and branched at the leading edge. To precisely define each filament at the branches, connected filaments must be segmented separately at the branching joints. Here, we fragmented each filament $i$ by the correlation of the 3D coordinates of each segment $j$. If the next coordinate $N+1$ cannot follow the tendency of the previous two coordinates $({V}_{\left(N,{N}-1\right)}^{i}=|{C}_{N}^{i}-\,{C}_{N-1}^{i}|)$ by 90 degree, the filament will be truncated and an additional filament will be generated from the break point ${{\deg }}({V}_{(N+1,N)}^{i}-\,{V}_{(N,N-1)}^{i}) \, < \, 90^\circ$.

Subsequently, we compared the polarity of each segment $({P}_{j}^{i})$ to the overall polarity of the filament $(S({F}^{i}))$. As actin filaments are in general short and straight within the tomogram, we define the overall polarity of the filament with the coordinates of the first and the last segment $(S({F}^{i})={C}_{{{{{{{\mathrm{last}}}}}}}}^{i}-{C}_{1}^{i})$. The segments following the overall polarity within 30 degrees were given a direction labeled $1$ and else $0$ $(\varepsilon {F}_{j}^{i})$;

1 $$\left\{\begin{array}{c}\,\varepsilon {F}_{j}^{i}=1{{{{{\rm{|}}}}}}S\left({F}^{i}\right)-{P}_{j}^{i}\le \,\pm \! 30^\circ \,\\ \varepsilon {F}_{j}^{i}=0{{{{{\rm{|}}}}}}S\left({F}^{i}\right)-{P}_{j}^{i} \, > \,\pm \! 30^\circ \end{array}\right.$$

The ${{{{{{\mathrm{mean}}}}}}}(\varepsilon {F}_{j}^{i})$ were used for the measurement of the filament polarity and the confidence of the polarity assignment was assessed based on $\varepsilon {F}_{j}^{i}$ (see further detail in ref. ^49^. Finally, only filaments passed the confidence test (combined confidence score $({ccs}({F}^{i}) \, > \, 0.6)$, Supplementary Fig. 4c) were then used for actin network analysis.

### Filament-membrane orientation

The cell membrane of each tomogram $t$ was manually segmented into $j$ segments $({M}_{j}^{t}=[{x,y,z}])$ and defined the local normal vectors ${N}_{j}^{t}$ by a plane-fit to the adjacent membrane points within a sphere with a radius of 40 nm. The normal vectors were then smoothed with the adjacent normal vectors $({{{{{{\mathrm{mean}}}}}}}({N}_{j}^{t}))$. We then fetched the closest membrane points for each actin segment. The membrane-segment orientation of each actin segment ${O}_{j}^{i}$ was then calculated by comparing the segment polarity and the average membrane normal vector $(O_{j}^{i}=({{{{{{\mathrm{mean}}}}}}}({N}_{j}^{t})-{P}_{j}^{i}))$. Finally, we averaged the membrane-segment orientation within a single filament to calculate its membrane-filament orientation ${{{{{{\mathrm{mean}}}}}}}({O}_{j}^{i})$.

For general categorization of the membrane-filament orientation, we defined the membrane-filament orientation into 3 intervals:

2 $$\left\{\,\begin{array}{c}{{{{{{\mathrm{mean}}}}}}}\left({O}_{j}^{i}\right)\le 80^\circ {{{{{\rm{|}}}}}}{{{{{{\mathrm{forward}}}}}}}\,{{{{{{\mathrm{filament}}}}}}}\\ 80^\circ < {{{{{{\mathrm{mean}}}}}}}\left({O}_{j}^{i}\right) \, < \, 100^\circ {{{{{\rm{|}}}}}}{{{{{{\mathrm{parallel}}}}}}}\,{{{{{{\mathrm{filament}}}}}}}\\ 100\le {{{{{{\mathrm{mean}}}}}}}\left({O}_{j}^{i}\right){{{{{\rm{|}}}}}}{{{{{{\mathrm{backward}}}}}}}\,{{{{{{\mathrm{filament}}}}}}}\end{array}\right.$$

### Quantification and statistical analysis

For categorization of different sub-domains of lamellipodia, the proportion of forward, parallel and backward filaments along the distance to the membrane were used (Fig. 4e–g). In every tomogram, the proportion of the three filament types was continuously calculated with 100 frames (frame size 40 nm). To reduce outliers effect, we used robust fit function in MATLAB for estimation of the proportion of the three filament types in every tomogram. The estimated filament proportion at the first frames in all the tomograms were then used for identifying different lamellipodia sub-domain. The portion of forward, parpallel, and backward filaments at the first frame were used as the coordinates of each tomogram for *k-means* clustering^80^. We clustered the tomograms into three classes. The combined filament proportions were directly used for continuous linear plotting (Fig. 4e–g). Histogram were normalized with the probability density function$({p}(x)=\,\frac{{{{{{{{\mathrm{count}}}}}}}}_{x}}{{{{{{{\mathrm{total}}}}}}\; {{{{{\mathrm{amount}}}}}}}})$ (Figs. 3b and 5). Continuous linear plots with error bars were plotted with the mseb function^81^ (Fig. 4e–g, Supplementary Fig. 5 and Supplementary Fig. 6d) and boxplots (Fig. 3c and Supplementary Fig. 6a–c) with the notBoxPlot function^82^ in MATLAB.

### Tomogram visualization

All isosurface visualization of actin filaments was rendered using UCSF Chimera or AMIRA. For visualization of actin filaments in Fig. 4b–d and Supplementary Fig. 3b and Supplementary Fig. 7, the refined 3D F-actin structure was used for representing. The refined 3D Arp2/3 structure was used in Supplementary Fig. 3b and Supplementary Fig. 7.

### Template matching and 3D averaging of Arp2/3

We used the Arp2/3-actin structure as a reference (EMD-4790^54^,) with a reported resolution at 32 Å for template matching procedure as described before^83^. For that purpose, we used 57 tomograms. The subtomograms were extracted with a box size of 31.8 nm. We then projected the subtomograms into 2D and performed 2D classification in Relion^79^. One thousand and four particles were selected and 3D averaged for the de novo 3D structure. We then expanded the data to 95 tomograms and performed template matching again with the new reference. After subtomogram projection and 2D classification, the selected 2D images were aligned to a template library generated by rotating the reference in 6° increment. We then performed 2D classification with restricted searching angle to 2° and mask around the mother filament and branch. The selected 2D images were then back-mapped to the tomograms and manually cleaned up by the reasonable particle coordinates in IMOD^84^. With a final 2D classification after the manual clean-up, 6149 particles were selected for 3D averaging in PyTom^83^.

### Statistics and reproducibility

We acquired 58 cryo-tomograms from 5 individual experiments and used 47 tomograms that contain a full set of images and low residual error. For actin filaments data analysis, 40 of the tomograms were eventually used for circumferential variability analysis. For Arp2/3 complex subtomogram averaging, additional 48 tomograms were acquired. The analysis are thoroughly described in the Materials and Methods section.

### Reporting summary

Further information on experimental design is available in the Nature Research Reporting Summary linked to this Article.

## Supplementary information

Supplementary Information

Description of Additional Supplementary Files

Supplementary Data File 1

Supplementary Movie 1

Supplementary Movie 2

Supplementary Movie 3

Reporting summary

## Data Availability

The EM structures of actin filament and Arp2/3 were uploaded on the Electron Microscopy Data Bank: EMD-15666 and EMD-15667. A tomogram of protruding, transition, and resting sub-domain were uploaded on the Electron Microscopy Data Bank: EMD-15644, EMD-15638, and EMD-15645. Supplementary Data File 1 contains tables for generating all plots.

## Code availability

The ATP code is available at https://github.com/WChung2/actin_polarity_toolbox.
