# Supplementary material for: A network of mixed actin polarity in the leading edge of spreading cells
Source: Commun Biol. 2022 Dec 7;5:1338. doi: 10.1038/s42003-022-04288-7 (PMC9727120; doi:10.1038/s42003-022-04288-7)
Supplement: Supplementary file 1 — Supplementary Information [file 42003_2022_4288_MOESM1_ESM.pdf]

A network of mixed actin polarity in the leading edge of spreading cells

Wen-Lu Chung[1], Matthias Eibauer[1], Wenhong Li[2], Rajaa Boujemaa-Paterski[1], Benjamin Geiger[2*] and Ohad Medalia[1*]

[1] Department of Biochemistry, University of Zurich, Winterthurerstrasse 190, 8057 Zurich, Switzerland

[2] Department of Immunology, and regenerative Biology, Weizmann Institute of Science, Rehovot 76100, Israel

Keywords: actin, cytoskeletal organization, cell adhesion, cryo-ET, Galectin-8

*Correspondence should be addressed to B.G. (benny.geiger@weizmann.ac.il) and O.M. (omedalia@bioc.uzh.ch)

Supplementary information

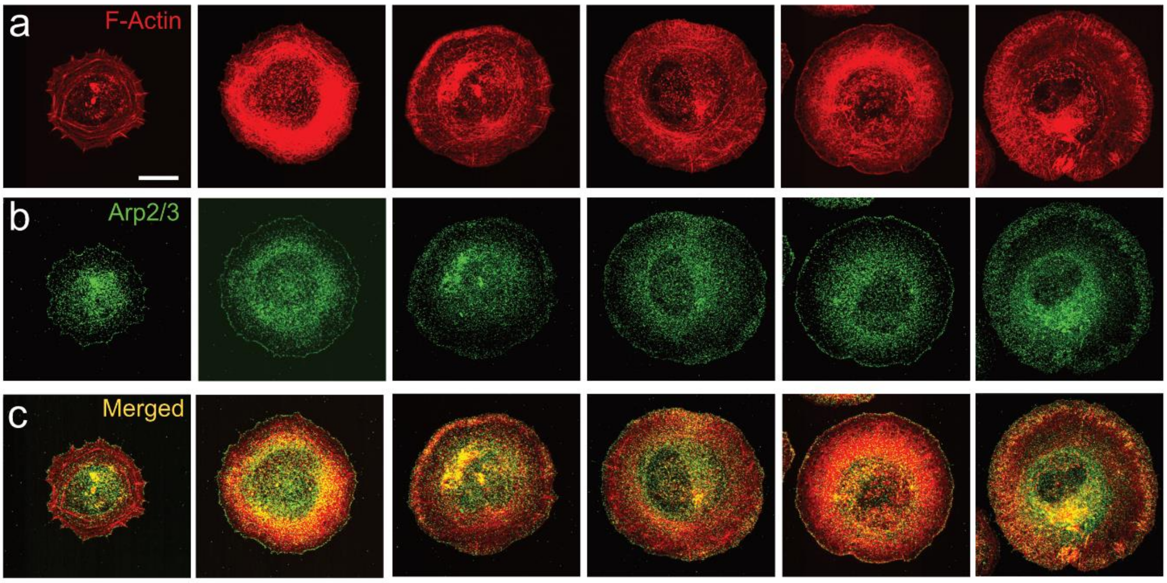

Supplementary Fig. 1 Lamellipodia in cells, spreading on Galectin-8. Immunofluorescent microscopy of MEFs spreading on Gal-8 coated substrate were acquired with spinning disk confocal microscopy. The Z-stack images were projected with ImageJ. The cells were chemically fixed 15 min after engaging to the Gal-8 coating glass. The six cells that are shown were stained with **a** phalloidin and **b** anti-p34-Arc. Merged color images are shown in **c.** Scale bar: 15 μm

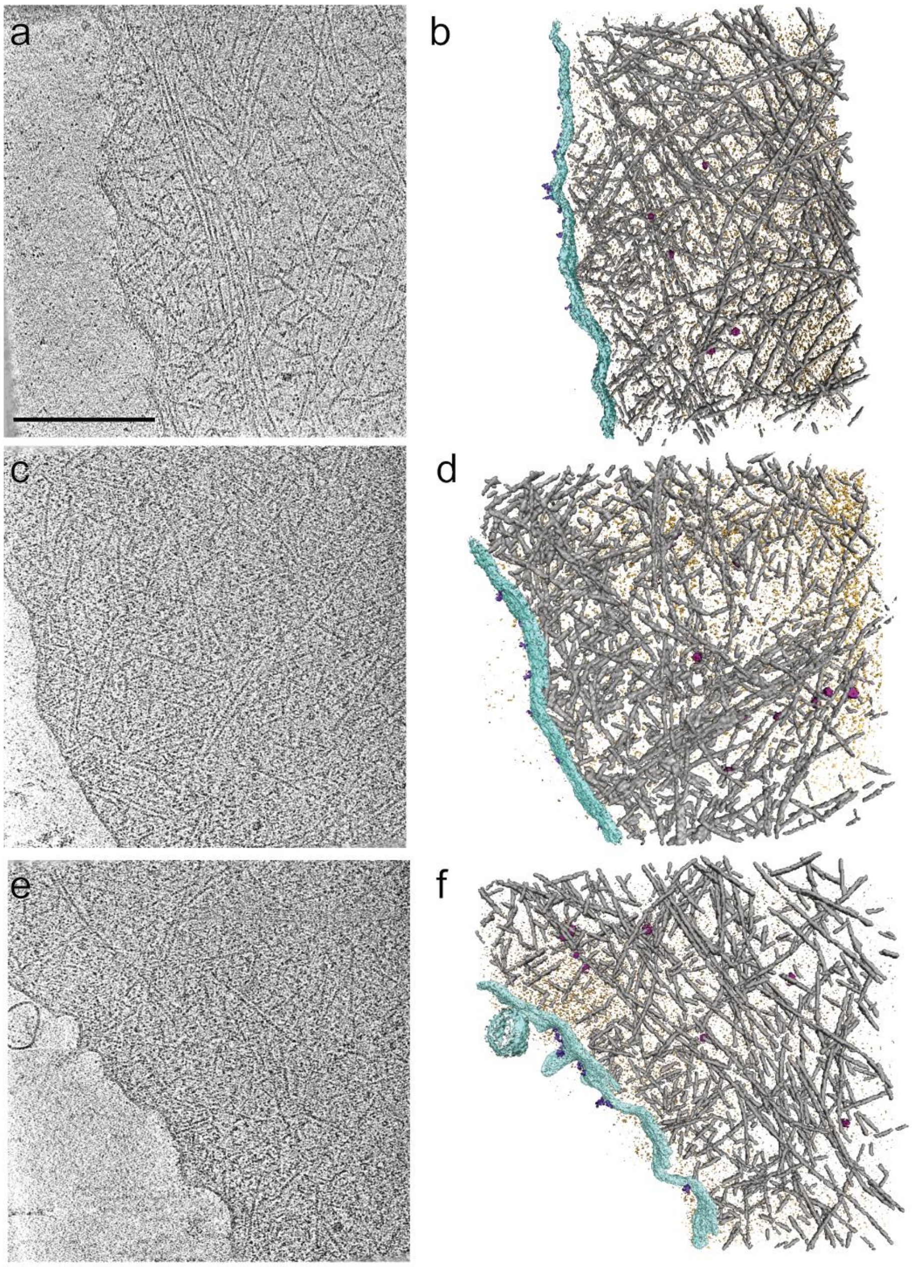

Supplementary Fig. 2 Cryo-tomograms of lamellipodia of MEFs spread on Gal-8. Cryo-tomograms of three cells are shown. x-y slices, 35.6 nm in thickness, through the tomograms **a, c, e** and the respective rendering isosurface views of the cryo-tomograms **b, d ,f**. Acin filaments (gray), membrane (turquoise), receptors (purple) and macromolecular complexes (dark red) are shown. Scale bar: 300 nm

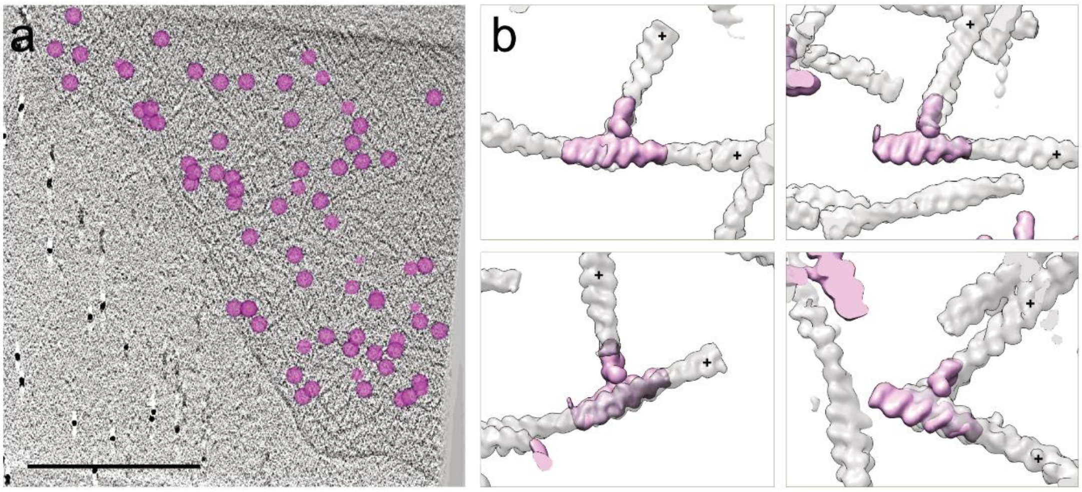

Supplementary Fig. 3 The localization of Arp2/3 identified by template matching. a The coordinates of the Arp2/3 (magenta) were superpositions on a 8.9 nm thick, x-y slice through the tomogram. Scale bar: 300 nm. **b** Surface rendered views of actin branches. Arp2/3 complexes are in magenta and actin filaments are in translucent gray. Barbed ends of daughter and mother filaments are marked with plus symbols.

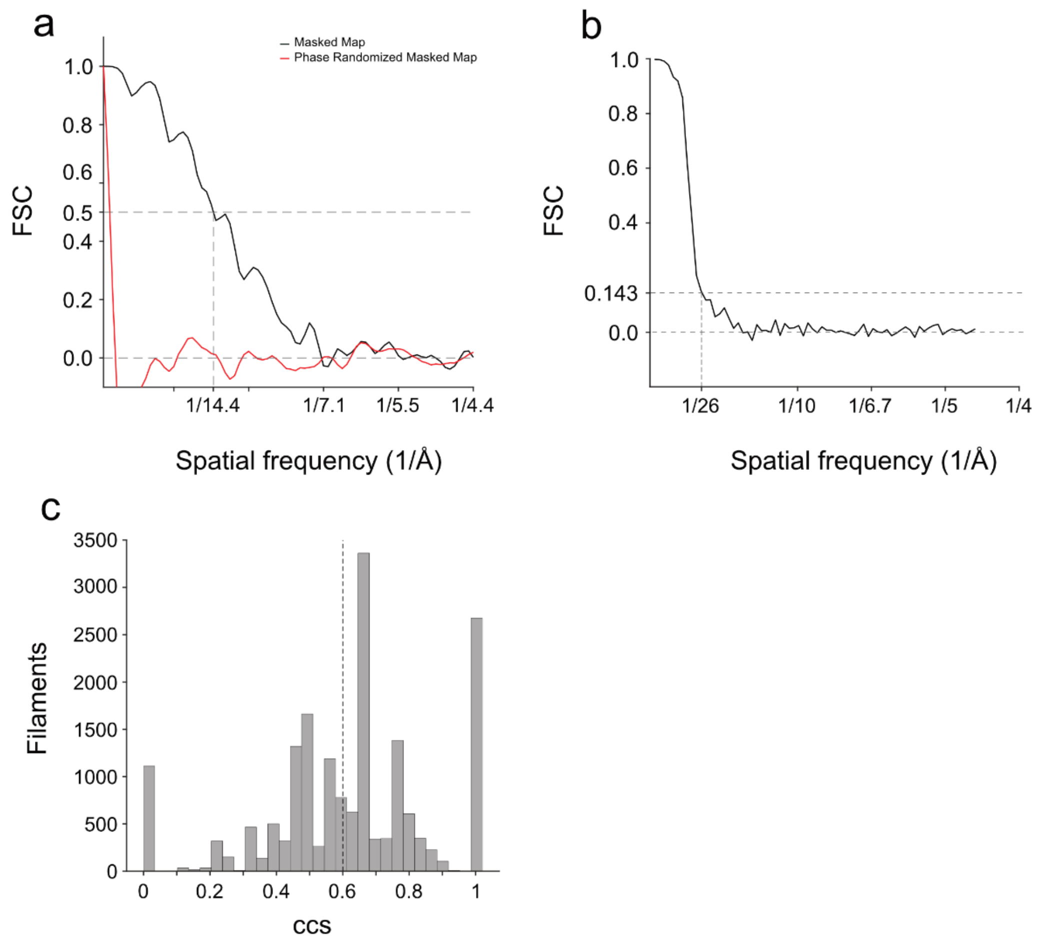

Supplementary Fig. 4. a. The refined actin structure in Fig. 2b and 2c shows spatial frequency of 1/14.4 Å indicated by 0.5 of Fourier shell correlation to EMD-15106. **b.** The refined Arp2/3 structure in Fig. 2e shows spatial frequency of 1/26 Å indicated by 0.143 gold-standard Fourier shell correlation criteria. **c.** Combined confidence score (ccs) of the acquired data described in method. ~70% of the filaments have passed the 0.6 ccs threshold (N = 47 tomograms).

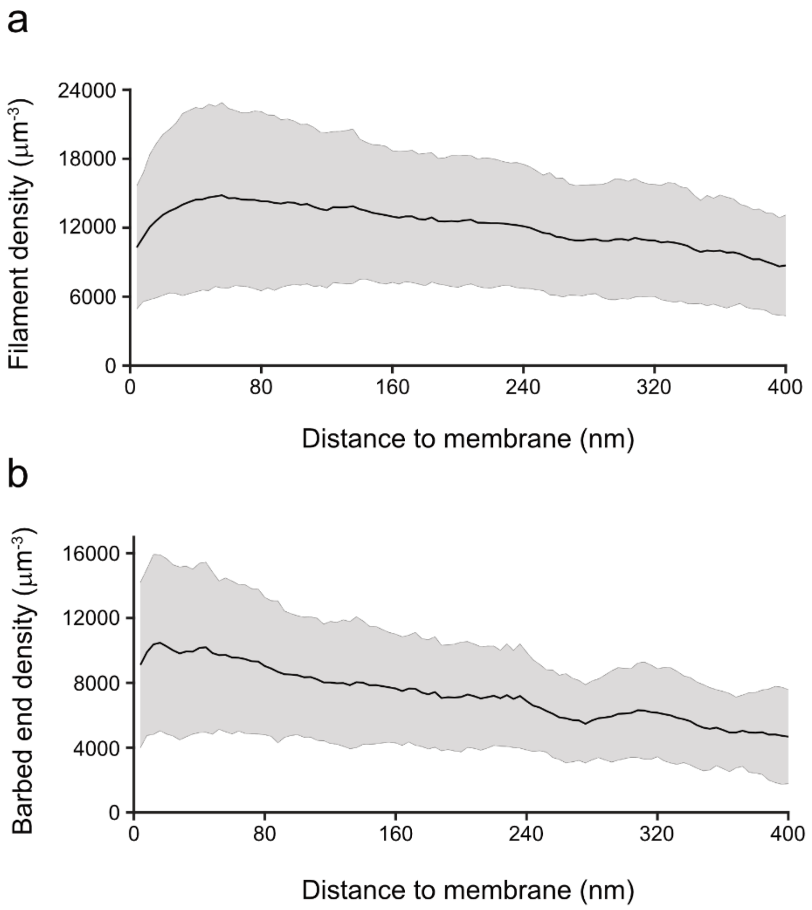

Supplementary Fig. 5. a Continuous filament density with shaded error bar of data standard deviation along the distance to the membrane (N = 38 tomograms). **b** Continuous barbed end density with shaded error bar of data standard deviation along the distance to the membrane (N = 39 tomograms).

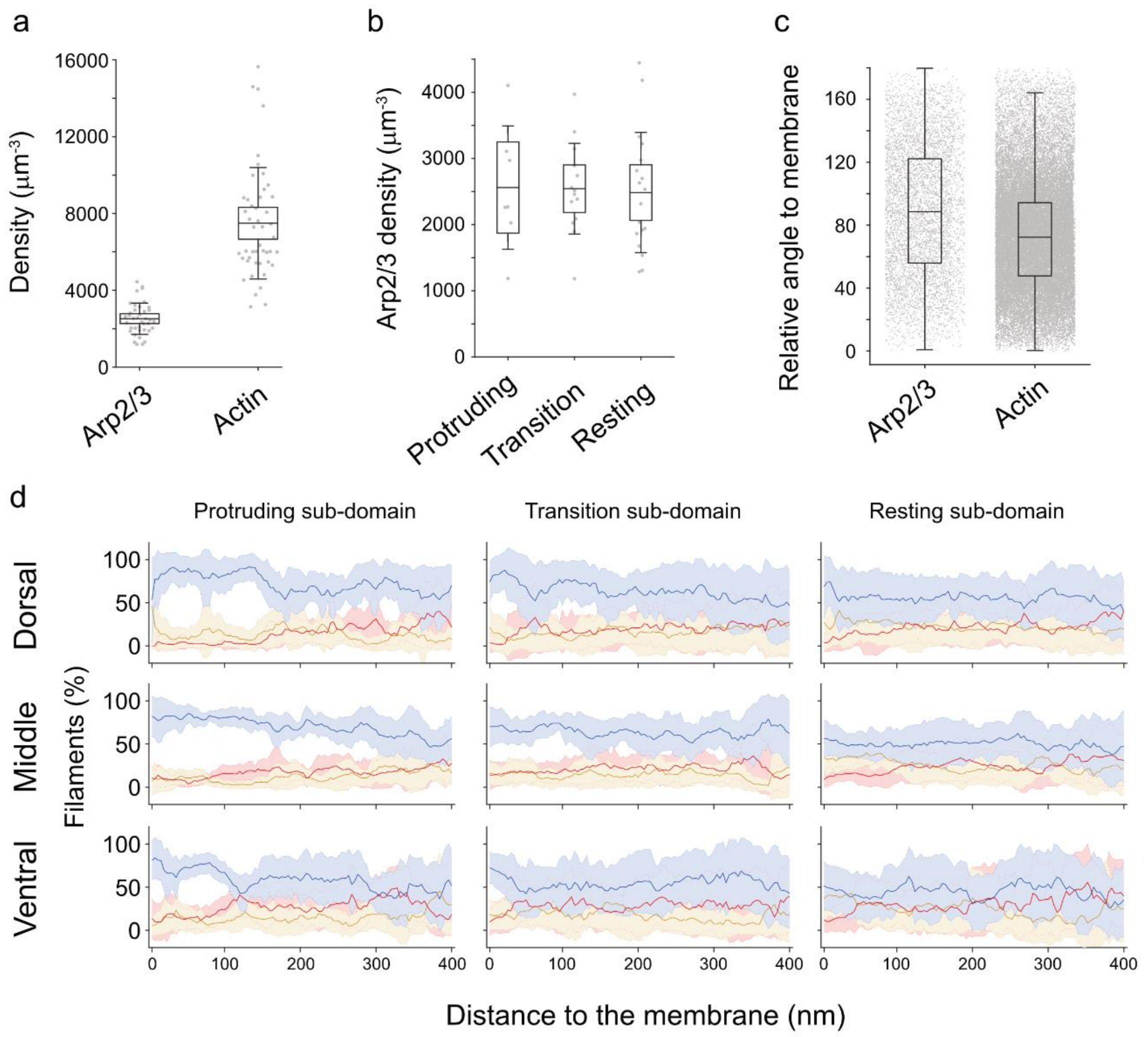

Supplementary Fig. 6. a. Boxplot shows the density of actin and Arp2/3 with 1 SD and 1.96 SEM in whiskers (N = 39, 47 tomograms, respectively). **b.** Boxplot shows the Arp2/3 density in three sub-domains with 1 SD and 1.96 SEM in whiskers (N = 7, 14, 18 tomograms, respectively). **c.** Boxplot shows the median and interquartile range of relative angle to membrane of actin filament and Arp2/3. Whiskers show the maximum and minimum. (N = 40 tomograms, Arp2/3 = 5630, Actin = 45104) **d**. The distribution of actin directionality along the thickness of the cell. The volumes were cut into three 30 nm sections, based on their proximity to the substrate (ventral: 0-30 nm, middle: 31-60 nm, and dorsal:61-90 nm) and the polarity of actin filaments were drowned as a function of distance from the cell edge. Forward (blue), parallel (mustard), and backward (red) actin orientations a are plotted as continuous line with a shaded error bar of data standard deviation, for protruding, transition, and resting sub-domains (N = 10, 16, 21 tomograms, respectively).

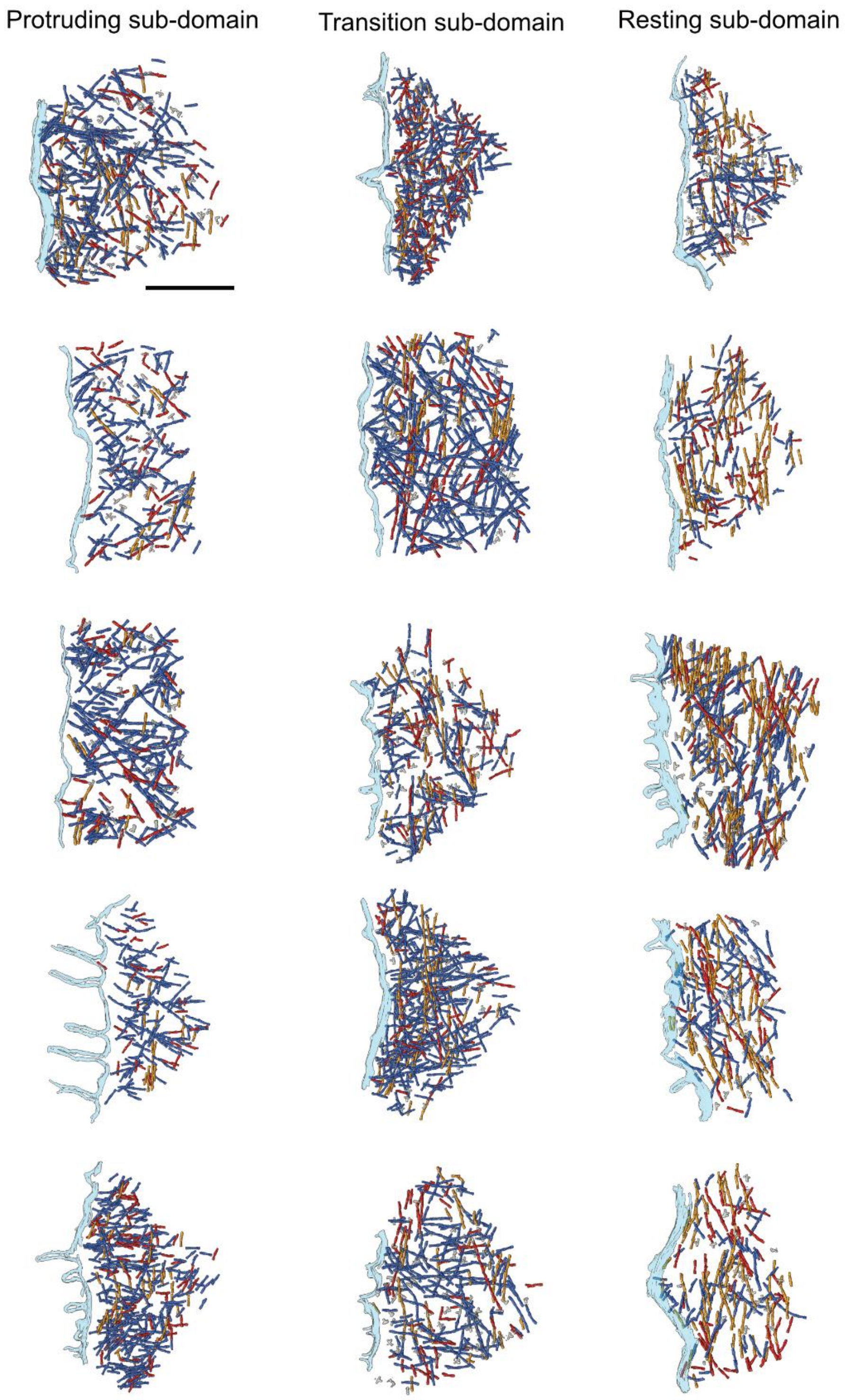

Supplementary Fig. 7. A collection of rendered tomograms, representing the 3 lamellipodial sub-domains**.** Arp2/3 are colored in gray. Scale bar: 300 nm

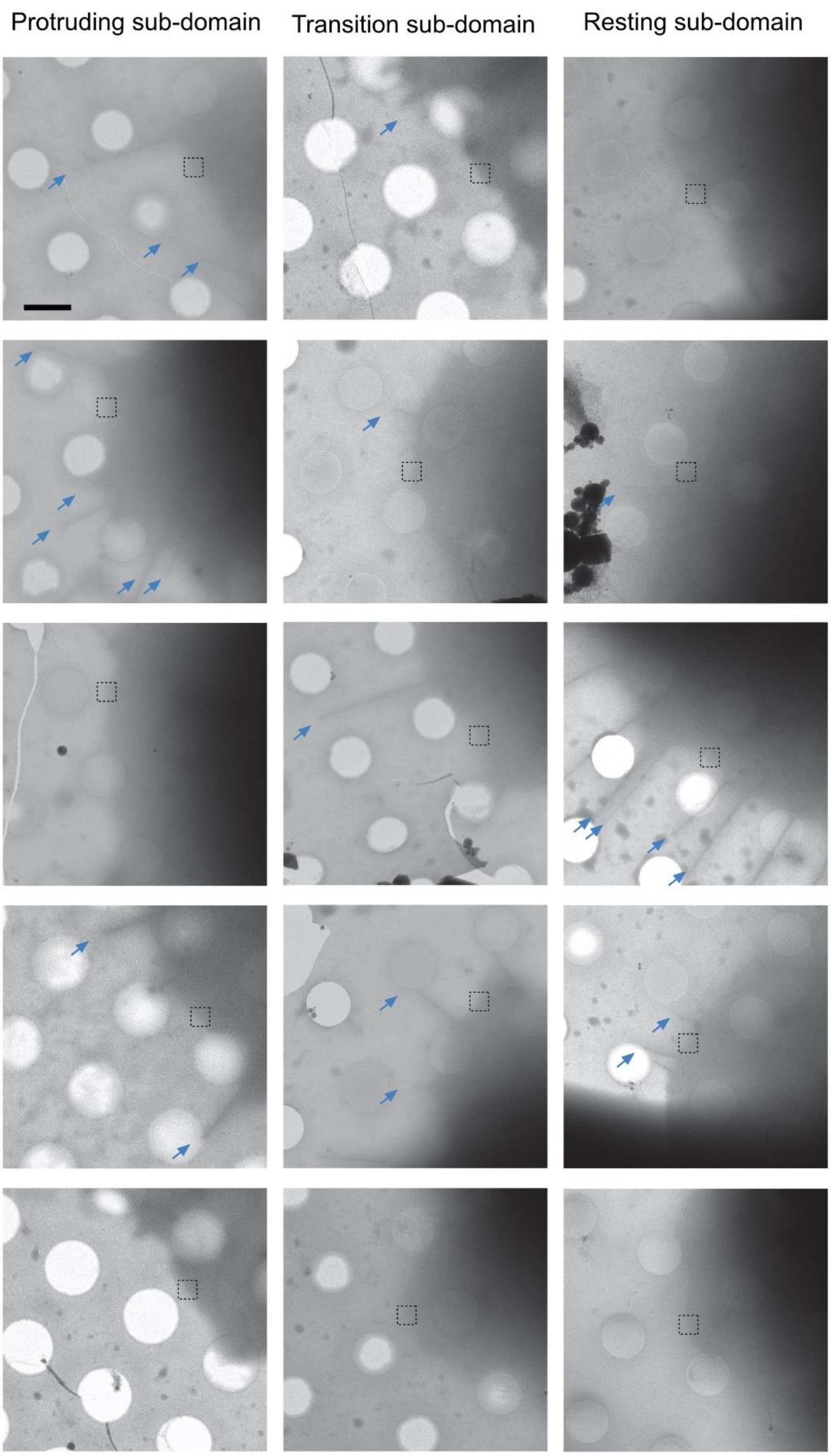

Supplementary Fig. 8. A collection of low magnification images of cell border. The box areas correlate to the data collected area in Supplementary Fig. 7. Filopodia are indicated with cyan arrows. Scale bar: 2 μm
