# Supplementary material for: A network of mixed actin polarity in the leading edge of spreading cells
Source: Commun Biol. 2022 Dec 7;5:1338. doi: 10.1038/s42003-022-04288-7 (PMC9727120; doi:10.1038/s42003-022-04288-7)
Supplement: Supplementary file 2 — Description of Additional Supplementary Files [file 42003_2022_4288_MOESM2_ESM.docx]

**Supplementary Movie 1.** A time-lapse series of a MEF cell spread on galectin-8 coated substrate, imaged by interference reflection microscopy.

**Supplementary Movie 2.** A time-lapse series of a MEF cell spread on galectin-8 coated substrate. The cell was transfected with Lifeact-mRuby.

**Supplementary Movie 3.** Scrolling through a cryo-tomogram of a MEF cell spread on a gal-8 coated EM grid. The tomogram corresponds to Fig. 3a, Scale bar: 300 nm.

**Supplementary Data** 1. An EXCEL file that contains the data that was used for generating Fig. 3,4,5 and Supplementary Fig. 4,5,6.
